# Macrophage migration inhibitory factor antagonist (p425) ameliorates kidney histopathological and functional changes in diabetic rats

**DOI:** 10.1590/2175-8239-JBN-2018-0184

**Published:** 2019-01-24

**Authors:** Jamal Khalilpour, Shiva Roshan-Milani, Farzaneh Hosseini Gharalari, Amin Abdollahzade Fard

**Affiliations:** 1 Urmia University of Medical Sciences Nephrology and Kidney Transplant Research Center Urmia Iran Urmia University of Medical Sciences, Nephrology and Kidney Transplant Research Center, Urmia, Iran; 2 Urmia University of Medical Sciences Faculty of Medicine Department of Physiology Urmia Iran Urmia University of Medical Sciences, Department of Physiology, Faculty of Medicine, Urmia, Iran; 3 Urmia University of Medical Sciences Emam Hospital Department of Pathology Urmia Iran Urmia University of Medical Sciences, Department of Pathology, Emam Hospital, Urmia, Iran; 4 Urmia University of Medical Sciences Neurophysiology Research Center Urmia Iran Urmia University of Medical Sciences, Neurophysiology Research Center, Urmia, Iran.

**Keywords:** Macrophage Migration-Inhibitory Factors, Diabetic Nephropathies, Macrophage Activation, Proteinuria, Glycosaminoglycans

## Abstract

**Introduction:**

It is hypothesized that increased macrophage migration inhibitory factor (MIF) expression may contribute to diabetic nephropathy (DN) pathogenesis. The aim of the present study was to investigate the renal effects of MIF inhibition in a diabetic experimental model.

**Methods:**

Eighteen male Wistar rats (230 ± 20 g) were divided into three groups: 1) control, 2) diabetic (STZ, 50 mg/kg, dissolved in saline, ip), 3) diabetic + MIF antagonist (p425, 1 mg/kg per day, ip, on the 21th day, for 21 consecutive days). The treatment started since we founwd a significant increase in urine albumin excretion (UAE) rate in the diabetic rats in comparison with the control rats. The rats were kept individually in metabolic cages (8 AM-2 PM) and urine samples were collected in the 21 and 42th day. At the end, blood and tissue samples were collected for biochemical (BS, UPE, urine GAG, BUN, Cr, Na, and K) and histological analyses.

**Results:**

The results of this study showed that MIF antagonist (p425) significantly decreased urine protein and GAG excretion, urine protein/creatinine ratio, and serum BUN and Cr in the streptozotocin-induced DN in the rats. Pathological changes were significantly alleviated in the MIF antagonist (p425)-administered DN rats.

**Conclusion:**

Collectively, these data suggested that MIF antagonist (p425) was able to protect against functional and histopathological injury in the DN.

## Introduction

Diabetic nephropathy (DN) is the *most common cause* of chronic kidney disease and is one of the *most important* long-term complications related to diabetes. Although the DN is conventionally viewed as a nonimmune disease, numerous evidence show that *inflammatory mechanism* may *play* a pivotal *role* in its development and progression.^1^^-^3

Several factors are involved in the development and progression of DN, including genetic factors, oxidative stress^4^ glomerular hyperfiltration,^5^ accumulation of advanced glycation end-products (AGEs),^6^ and overexpression of transforming growth factor-b (TGF-b), followed by increase of extracellular matrices.^7^

The glomerular basement membrane (GBM) mainly consists of laminin, type IV collagen, and heparan sulfate (HS) proteoglycans (HSPGs). Degradation of these components results in breakdown of the basement membrane structure. Heparan sulfate proteoglycans (HSPGs) are abundant in extracellular matrices (ECMs), including basement membranes, and consist of diverse core polypeptides and HS.^8^^,^^9^

HS maintains the mechanical integrity of glomerular basement membranes. Direct *heparitinase* digestion through heparitinase existing in glomerular basement membranes results in a loss of membrane function.^10^ In patients with DN, loss of HSPG in glomerular extracellular matrices has been reported.^11^ Both the urinary and plasma levels of heparanase have been reported to be elevated in type 2 diabetes. In DN, an increase in urinary heparanase and its activity as an endoglycosidase that specifically cleaves HS in side chains of HSPG is observed in both type 1 and type 2 diabetic patients with proteinuria.^12^^,^^13^ Therefore, loss of the HS in the glomerular basement membrane *results* in a decrease of the anionic charge barrier and may possibly be one of the major causes of albuminuria in the DN.^14^^,^^15^

Inflammatory cells, mainly macrophages, are present in the glomeruli and interstitium of patients with the DN, suggesting that the inflammatory process is also involved in the development of DN.^16^^,^^17^ Heparanase activity has been reported in macrophages, platelets, neutrophils, monocytes, Langerhans cells, *and many other* cells.^18^^-^23 It is assumed that secreted or membrane-associated heparanase is responsible for the degradation of ECM. Macrophage migration inhibitory factor (MIF) is the first molecule to arrive at the inflammation site and likely determines the degree of cellular inflammation.^24^ The MIF has been involved in both types of diabetes,^25^ and there is evidence linking the MIF with DN. Moreover, the MIF also increases in experimental DN^26^ before the onset of microalbuminuria.^27^ It is hypothesized that increased MIF expression may contribute to DN pathogenesis. In the present study, we investigated the renal effects of MIF inhibition in a diabetic experimental model.

## Material and methods

### Experimental design

Eighteen male 10-*week*-*old* Wistar rats weighing (230 ± 20 g) were purchased from the animal house of the Urmia University of Medical Sciences, Urmia, Iran. All procedures for the animals were conducted in accordance with the Principles of Laboratory Animal Care (NIH publication no. 85-23, revised 1985) and approved by the Ethical Committee of the Urmia University of Medical Sciences. The animals were maintained under controlled conditions of temperature (21 ± 2ºC) and a 12/12 h light/dark cycle. The animals were fed normal rat diet and water. The animals were randomly divided into three groups (six animals each): Group 1 - healthy control (0.2 mL ip injection of normal saline), Group 2 - diabetic group, and Group 3 - diabetic group treated with MIF antagonist (p425, 1 mg/kg; daily, ip).

In the diabetic group animals, diabetes was induced by a single intraperitoneal injection of streptozotocin (STZ, 50 mg per kg body weight, dissolved in saline), while the control rats were injected only with normal saline. Five days after the STZ injection, fasting blood glucose levels were determined with a glucose strip test in a glucometer. Rats with blood glucose levels above 200 mg/dL were defined as the diabetic animals. MIF inhibitor (p424) was dissolved in normal saline. The treatment started 21 days after the STZ injection and this was considered the first day of treatment. The treatment was continued daily for three weeks.

From the beginning of the third week after the induction of diabetes, the rats were kept individually in metabolic cages (8 AM-2 PM) and urine samples were collected for 6-h measurement of urine protein excretion (UPE) and urine creatinine. The results revealed a significant increase in the urine albumin excretion (UAE) rate in the diabetic rats in comparison with the control rats and the animals were considered nephropathic.

At the end of the sixth week, the rats were kept individually in metabolic cage (8 AM-2 PM) and 6-h urine samples were collected for biochemical analysis. Then, six rats from every investigated group were sacrificed under ether anesthesia. Moreover, blood and tissue samples were collected.

### Biochemical analyses

Blood samples were collected by cardiac puncture for measurement of Na, K, BUN, and creatinine (Cr). Serum BUN and creatinine were measured using auto-analyzer and serum Na and K concentrations were measured by flame photometry. Moreover, 6-h urine samples were collected for measurement of BUN, Cr, UPE, and glycosaminoglycan (GAG).

Urine protein excretion (UPE) was determined by a kit (Pars Azmon, Iran) and UPE was measured by quantitative reaction with bromocresol green^28^ using bovine serum albumin as standard. A volume of 10 µL of the sample and standard were mixed separately with 1 mL of bromocresol green and then the absorbance was read at 625 nm.

Urinary GAG was measured spectrophotometrically at a wavelength of 520 nm in 6-h urine samples, with a colorimetric method described by Jong,^29^ using 1.9 dimethylene blue and bovine kidney heparan sulfate as standard (Sigma Cat No H7640).

### Histological study

Following the blood sample collection, the right kidney was removed and stored in 10% formaldehyde. Kidney sections (5 µm) were stained with periodic acid-Schiff (PAS) and Masson's trichrome (MTC) for histologic and morphometric analysis. Mesangial matrix accumulation was assessed by PAS-positive staining in nuclei-free areas of the mesangium. Mesangial matrix was evaluated in 30 randomly selected glomeruli and scored in a blinded manner on a scale of 0 to 4, where 0 = 0-5%, 1= 0.5-25%, 2 = 0.25-50%, 3 = 0.50-75%, and 4 = 0.75% deposition. The scores revealed variations in the extent rather than intensity of staining.^30^ The 'sclerotic index' referred to the mean score. Collagen deposition was measured with Masson's trichrome staining of 30 glomeruli, scored in a blinded manner using the above-mentioned system and reported as an arbitrary unit.^30^^,^^31^ Then, it was reported as mean sclerotic index and glomerular collagen staining score for each group.

### Statistical analysis

Data are reported as the mean ± SD. Statistical significance of differences was assessed with one-way ANOVA on SPSS (Version 18; SPSS Inc., Chicago, USA) followed by Tukey's test. A p value less than 0.05 was considered statistically significant. Linear regression analyses were applied to evaluate the relationship between the two variables.

## Results

### Effects p425 on serum BUN, creatinine, K and Na

As shown in Table 1, a significant increase could be noted in the serum BUN, Cr, and K levels of the DN rats in comparison with the control (*p* < 0.001, *p* < 0.001, and *p* < 0.05, respectively). However, there was no significant difference in the serum concentration of Na between the groups. Moreover, the administration of MIF antagonist (p425) in the DN group reduced serum BUN and Cr and these changes were statistically significant (*p* < 0.01 and *p* < 0.05, respectively). Collectively, these data suggested that p425 was able to protect against DN injury.

**Table 1:** Serum levels of creatinine, BUN, sodium and potassium in studied groups

Group	Creatinine (mg/dl)	BUN (mg/dl)	Na (meq/L)	K (meq/L)
Control	0.45 ± 0.1	13.3 ± 2.3	139.5 ± 4.8	4.2 ± 0.4
DN	1.04 ± 0.2*	26.1 ± 4*	141.2 ± 3.2	5.3 ± 0.6*
DN +p425	0.61 ± 0.1^†^	18.2 ± 1.9^†^	139 ± 4.7	4.6 ± 0.5

The values are shown as mean ± SD. DN (diabetic nephropathy), p425 (MIF antagonist)* and ^†^indicate significance in comparison with control and DN group, respectively (*p*<0.05).

### Effects of p425 on blood glucose

**Figure 1:**
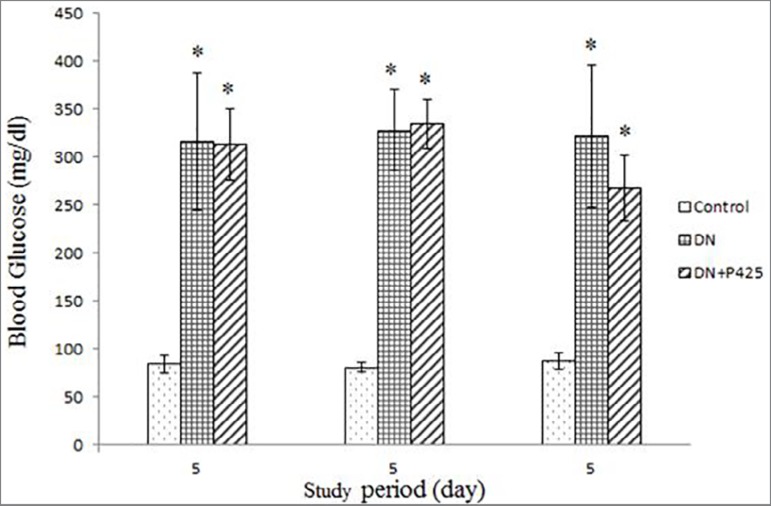
Blood glucose in the 5, 21, 42th day of the study period. The values are shown as mean ± SD. DN: Diabetic Nephropathy.; p425: MIF antagonist. *indicated significance in comparison with the control (*p* < 0.001).

As shown in Figure 1, in the 5, 21, and 42th day of the 6-week study period, blood glucose levels were 84.4 ± 8.9, 81.2 ± 5.1, and 87.7 ± 8.7 mg/dL, respectively, for healthy controls while the DN animals had significantly elevated levels (316.4 ± 72.1, 328.1 ± 42.3 and 321.6 ± 74.3 mg/dL, respectively, *p* < 0.001). The elevated blood glucose level remained stable over time. The administration of p425 in the DN group in the 42th day study period reduced blood glucose in comparison with the DN group without p425, though this change was not statistically significant (Figure 1).

### Effects p425 on urine GAG

At the end of the 6-week study period, urinary 6-hour GAG levels were significantly higher in the DN rats in comparison with the healthy controls (*p* < 0.001). However, the MIF antagonist (p425) group had lower GAG excretion than the untreated DN rats; this change was statistically significant (*p* < 0.001, Table 2).

**Table 2:** Urine levels of creatinine, protein, and glycosaminoglycan (GAG)

Group	Cr U (mg/dl)	Prot. U 21 (mg/6h)	Prot. U 42 (mg/6h)	GAG (mg/6h)
Control	33.9 ± 4.8	8.8 ± 2.4	9.4 ± 2.6	41.6 ± 6.8
DN	26.9 ± 7.2	50 ± 14.6*	70 ± 2.4*	131.2 ± 19.2*
DN +p425	29.4 ± 7.7	48.6 ± 18.3*	17.4 ± 8.4^†^	60.8 ± 12^†^

The values are shown as mean ± SD. DN (diabetic nephropathy), p425 (MIF antagonist) Uprot.21 and 42 (urine protein in 21th and 42th day). * and ^†^ indicate significance in comparison with control and DN group, respectively (*p* < 0.01).

### Effects of p425 on urine protein excretion and protein-creatinine ratio (PCR):

**Figure 2:**
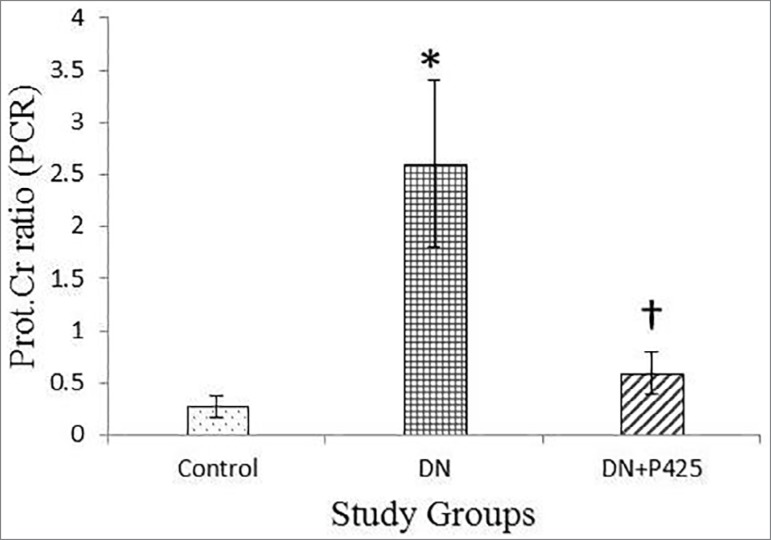
Urine protein/creatinine ratio. The values are shown as mean ± SD. DN: Diabetic Nephropathy; p425: MIF antagonist; * and † indicate the significance in comparison with the control and DN group, respectively (*p* < 0.001).

At the end of the 3-week study period, urinary 6-hour protein excretion level was 8.8 ± 2.4, 50 ± 14.6 and 48.6±18 mg/6h in the healthy control, diabetic control, and treated diabetic control, respectively. Urinary protein excretion increased significantly in the diabetic rats (*p* < 0.01). Moreover, urinary 6-hour protein excretion level remained high over time in the DN in comparison with the control (*p* < 0.001). However, the administration of MIF antagonist (p425) significantly reduced urine 6-h protein excretion (*p* < 0.001) in the DN in comparison with the untreated DN group (Table 2). The results indicated an improvement effect of p425 on the STZ-induced *DN* in the rats, which was evidenced by a *significant* decrease (*p* < 0.001) in the urinary protein/creatinine ratio (Figure 2).

### Correlation between urinary protein and urinary glycosaminoglycan excretion

**Figure 3:**
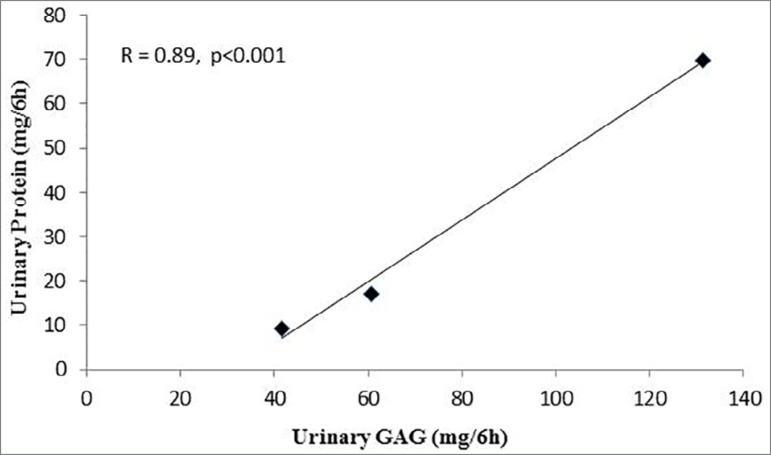
Linear regression curves representing urinary protein and glycosaminoglycan in the normal and diabetic rats (r= 0.89 and *p* < 0.001).

Urinary protein excretion was significantly correlated with urinary glycosaminoglycan excretion (r =0.89, *p* < 0.001) in all the study groups (Figure 3).

### Effects of p425 on morphological change after DN injury

**Figure 4:**
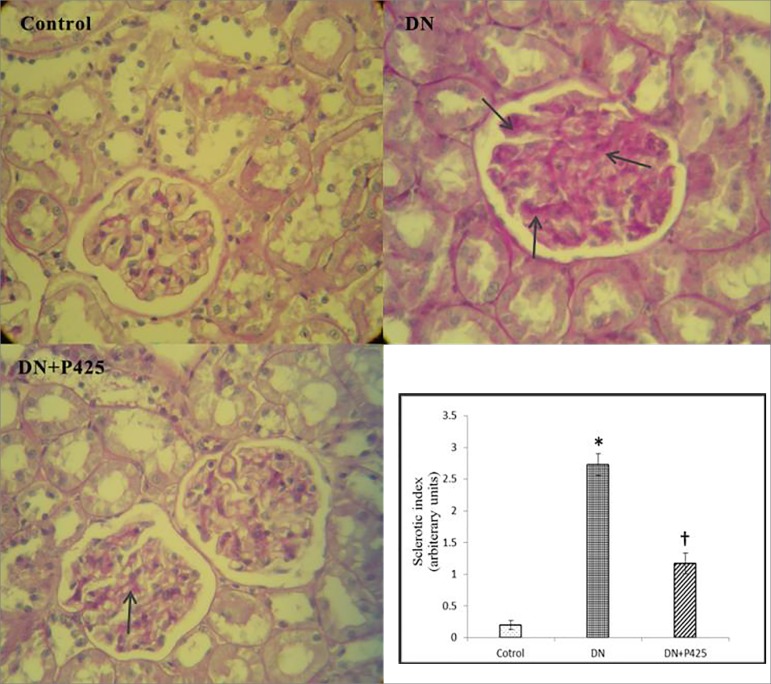
Photomicrographs of PAS staining of renal tissues. DN: Diabetic Nephropathy; p425: MIF antagonist. Arrows show the mesangial matrix accumulation in nuclei-free areas of the mesangium in glomeruli. The "sclerotic index" refers to the mean ± SE score. The scores reflect variations in the extent rather than intensity of staining. * and † indicate significance in comparison with the control and DN group, respectively (*p* < 0.001). Magnification: 400×.

**Figure 5:**
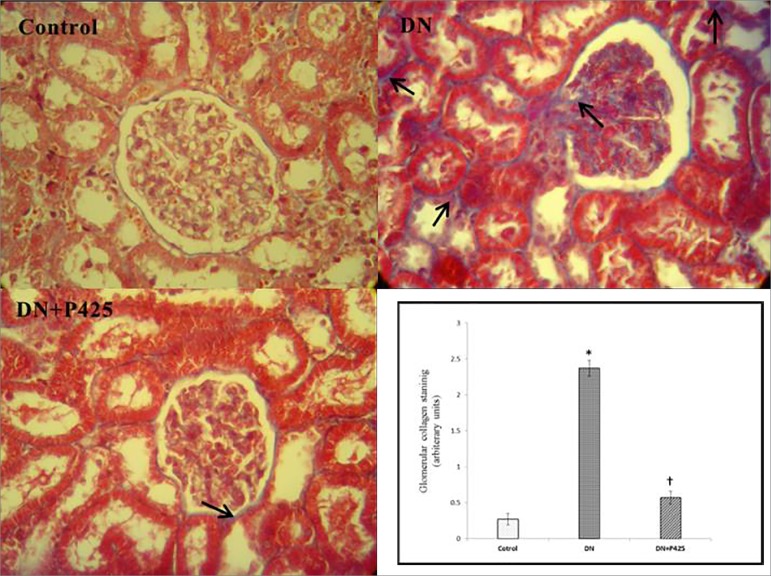
Photomicrographs of Masson trichrome (MTC) staining of renal tissues. DN: Diabetic Nephropathy; p425: MIF antagonist. Arrows show the collagen deposition. The "glomerular collagen deposition" refers to the mean ± SE score. * and † indicate significance in comparison with the control and DN group, respectively (*p* < 0.001). Magnification: 400×.

As shown in Figures 4 and 5, DN led to tubular injury characterized by pronounced renal tubular detachment, tubular cell necrosis, and loss of brush border as well as increased glomerular surface area, mesangial expansion, thickening of the GBM and Bowman's capsule, and increased deposition of matrix proteins within the mesangial matrix. The 'sclerotic index', which reflects glomerular matrix accumulation, increased in non-treated DN rats in comparison with the healthy control (2.73 ± 0.17 vs. 0.2 ± 0.07, three weeks after onset of albuminuria, *p* < 0.001), but was significantly reduced (1.17 ± 0.16, *p* < 0.001) with p425 treatment (Figure 4). Collagen deposition in the glomerulus was markedly reduced in the DN group following p425 treatment (2.37 ± 0.11 vs. 0.57 ± 0.09, *p* < 0.001) (Figure 5). As shown in Figures 4 and 5, the aforementioned pathological changes were significantly alleviated in the p425 administered-DN rats.

## Discussion

The results of the present study demonstrated that MIF antagonist (p425) reduced proteinuria and urine GAG excretion and prevented glomerular basement membrane thickening and ECM accumulation in the STZ induced DN rats. Over the 6-week study, there was a significant increase in urinary protein excretion. Glomerular abnormalities included thickening of the GBM. Changes in heparan sulfate GAG chains were not investigated in the present study; however, a loss of heparan sulfate proteoglycans in the GBM contributed to proteinuria in glomerular diseases including the DN.^32^ The results of the present study confirmed that urinary GAG excretion was elevated in the non-treated diabetic rats as shown before in diabetic rats by Reddi.^33^^,^^34^ A significant decrease in urinary GAG excretion was detected in the p425-treated DN rats compared with the non-treated DN rats. Urinary GAG excretion was significantly correlated with protein excretion in all the study groups. This might indicate that increased loss of proteoglycans from diabetic kidneys is prevented by the MIF antagonist (p425) treatment. This is the first report of such an effect.

Results of biochemical parameters are in agreement with light microscopy findings. These findings suggest that MIF inhibition may be beneficial to DN and this effect may be attributable to its modulation of macrophage activation as well as glomerular basement membrane. We are interested in the MIF as a potential therapeutic target for DN not only because it is elevated in DN patients and animal models^26^^,^^27^ but also due to its pivotal role in inflammation cascade and macrophage polarization. The MIF is the first molecule to arrive at the inflammation site and likely determines the degree of cellular inflammation.^24^

Although heparanase activity changes were not examined in the present study, heparanase activity has been reported in macrophages, platelets, neutrophils, monocytes, Langerhans cells, *and many other* cells.^18^^-^23 It was assumed that secreted or membrane-associated heparanase was responsible for the degradation of *the* ECM. Heparanase activity, an endoglycosidase that specifically cleaves HS side chains of HSPG, has been observed in both type 1 and type 2 diabetic patients with proteinuria.^12^^,^^13^ Therefore, loss of HS in the glomerular basement membrane *results* in a decrease of the anionic charge barrier and may possibly be one of the major causes of proteinuria in DN.

DN is characterized by progressive fibrosis as a final pathway, which eventually affects all substructures of the kidney. Recent studies have revealed interstitial infiltration of macrophages that produce cytokines responsible for histological injury and fibroblast proliferation and activation.^35^^,^^36^ Some studies have demonstrated that classically activated M1 macrophages induce podocyte permeability.^37^ The present study provided evidence that the inhibition of the MIF was functionally significant, which may have resulted in reduced macrophage activation in the diabetic kidney, associated with reduced proteinuria, ECM accumulation, and collagen deposition in glomeruli in vivo. These effects may be attributable to the inhibitory effect of the MIF on macrophage activation in the diabetic kidney. Thus, MIF inhibition may be a potential therapeutic strategy for DN.

In conclusion, the MIF antagonist (p425) treatment reduces urinary protein and GAG excretion, prevents GBM thickness and collagen deposition, and probably protects from the loss of GBM anionic content in DN rats. Preservation of GAG and anionic charges of the GBM seems to be one of the mechanisms by which p425 mitigates the proteinuria in diabetic rats.

## References

[1] Mora C, Navarro JF. Inflammation and diabetic nephropathy. Curr Diab Rep. 2006;6(6):463-8. Epub 2006/11/23. 10.1007/s11892-006-0080-1 17118230

[2] Tuttle KR. Linking metabolism and immunology: Diabeticnephropathy is an inflammatory disease. Journal of the AmericanSociety of Nephrology: JASN. 2005;16(6):1537-8. 10.1681/ASN.2005040393 15872083

[3] Fard AA, Abbasnezhad P, Makhdomi K, Salehi M, Karamdel HR, Saboory E. Association of Serum Prolactin Concentrations with Renal Failure in Diabetic Patients. Romanian Journal of Diabetes Nutrition and Metabolic Diseases. 2017;24(3):179-85.

[4] Zemin C, EC. M. Pathogenesis of diabetic nephropathy. J Diabetes Invest 2011;2:243-7. 10.1111/j.2040-1124.2011.00131.x PMC4014960 24843491

[5] Magee GM, Bilous R, Cardwell CR, Hunter SJ, Kee F, Fogarty DG. Is hyperfiltration associated with the future risk of developing diabetic nephropathy? A meta-analysis. Diabetologia. 2009;52(4):691-7. 10.1007/s00125-009-1268-0 19198800

[6] Brownlee M, Cerami A, Vlassara H. Advanced glycosylation end products in tissue and the biochemical basis of diabetic complications.. N Engl j Med. 1988;318:1315-21. 10.1056/NEJM198805193182007 3283558

[7] Ziyadeh FN, Sharma K, Ericksen M, Wolf G. Stimulation of collagen gene expression and protein synthesis in murine mesangial cells by high glucose is mediated by autocrine activation of transforming growth factor-beta. Journal of Clinical Investigation. 1994;93(2):536. 10.1172/JCI117004 PMC293875 8113392

[8] Whitelock JM, Murdoch AD, Iozzo RV, Underwood PA. The degradation of human endothelial cell-derived perlecan and release of bound basic fibroblast growth factor by stromelysin, collagenase, plasmin, and heparanases. Journal of Biological Chemistry. 1996;271(17):10079-86. 10.1074/jbc.271.17.10079 8626565

[9] Kato M, Wang H, Kainulainen V, Fitzgerald ML, Ledbetter S, Ornitz DM, et al. Physiological degradation converts the soluble syndecan-1 ectodomain from an inhibitor to a potent activator of FGF-2. Nature medicine. 1998;4(6):691-7. 10.1038/nm0698-691 9623978

[10] Kanwar YS, Farquhar MG. Presence of heparan sulfate in the glomerular basement membrane. Proceedings of the National Academy of Sciences. 1979;76(3):1303-7. 10.1073/pnas.76.3.1303 PMC383239 155819

[11] Makino H, Ikeda S, Haramoto T, Ota Z. Heparan sulfate proteoglycans are lost in patients with diabetic nephropathy. Nephron. 1992;61(4):415-21. 10.1159/000186959 1501738

[12] Shafat I, Ilan N, Zoabi S, Vlodavsky I, Nakhoul F. Heparanase levels are elevated in the urine and plasma of type 2 diabetes patients and associate with blood glucose levels. PloS one. 2011;6(2):e17312. 10.1371/journal.pone.0017312 PMC3043098 21364956

[13] Rops AL, van den Hoven MJ, Veldman BA, Salemink S, Vervoort G, Elving LD, et al. Urinary heparanase activity in patients with Type 1 and Type 2 diabetes. Nephrology Dialysis Transplantation. 2011;27(7):2853-61. 10.1093/ndt/gfr732 22187315

[14] Kolset S, Reinholt F, Jenssen T. Diabetic nephropathy and extracellular matrix. Journal of Histochemistry & Cytochemistry. 2012;60(12):976-86. 10.1369/0022155412465073 PMC3527883 23103723

[15] Mansouri E, Panahi M, Ghaffari MA, Ghorbani A. Grape seed proanthocyanidin extract ameliorates albuminuria and renal sclerosis in experimental diabetic nephropathy rats. Asian Biomedicine. 2012;6(2):195-202.

[16] Furuta T, Saito T, Ootaka T, Soma J, Obara K, Abe K, et al. The role of macrophages in diabetic glomerulosclerosis. American Journal of Kidney Diseases. 1993;21(5):480-5. 10.1016/s0272-6386(12)80393-3 8488815

[17] Shikata K-I, Makino H. Role of macrophages in the pathogenesis of diabetic nephropathy. Type-2 Diabetic Nephropathy in Japan: Karger Publishers; 2001. p. 46-54. 10.1159/000060147 11665287

[18] Freeman C, Parish RC. Human platelet heparanase: purification, characterization and catalytic activity. Biochemical Journal. 1998;330(3):1341-50. 10.1042/bj3301341 PMC1219281 9494105

[19] Matzner Y, Bar-Ner M, Yahalom J, Ishai-Michaeli R, Fuks Z, Vlodavsky I. Degradation of heparan sulfate in the subendothelial extracellular matrix by a readily released heparanase from human neutrophils. Possible role in invasion through basement membranes. Journal of Clinical Investigation. 1985;76(4):1306. 10.1172/JCI112104 PMC424062 2997275

[20] Sewell RF, Brenchley PE, Mallick NP. Human mononuclear cells contain an endoglycosidase specific for heparan sulphate glycosaminoglycan demonstrable with the use of a specific solid-phase metabolically radiolabelled substrate. Biochemical Journal. 1989;264(3):777-83. 10.1042/bj2640777 PMC1133653 2533499

[21] Naparstek Y, Cohen IR, Fuks Z, Vlodavsky I. Activated T lymphocytes produce a matrix-degrading heparan sulphate endoglycosidase. Nature. 1984;310(5974):241-4. 10.1038/310241a0 6205275

[22] Marchetti D, Li J, Shen R. Astrocytes contribute to the brain-metastatic specificity of melanoma cells by producing heparanase. Cancer Research. 2000;60(17):4767-70. 10987284

[23] Bernard D, MÃ©hul B, Delattre C, Simonetti L, Thomas-Collignon A, Schmidt R. Purification and characterization of the endoglycosidase heparanase 1 from human plantar stratum corneum: a key enzyme in epidermal physiology? Journal of investigative dermatology. 2001;117(5):1266-73. 10.1046/j.1523-1747.2001.15401.x 11710943

[24] Malorny U, Goebeler M, Gutwald J, Roth J, Sorg C. Differences in migration inhibitory factor production by C57B1/6 and BALB/c mice in allergic and irritant contact dermatitis. International Archives of Allergy and Immunology. 1990;92(4):356-60. 10.1159/000235164 2083971

[25] SÃ¡nchez-Zamora YI, Rodriguez-Sosa M. The role of MIF in type 1 and type 2 diabetes mellitus. Journal of diabetes research. 2014;2014. 10.1155/2014/804519 PMC3910331 24527464

[26] Chow F, Ozols E, Nikolic-Paterson DJ, Atkins RC, Tesch GH. Macrophages in mouse type 2 diabetic nephropathy: correlation with diabetic state and progressive renal injury. Kidney international. 2004;65(1):116-28. 10.1111/j.1523-1755.2004.00367.x 14675042

[27] Watanabe T, Tomioka NH, Doshi M, Watanabe S, Tsuchiya M, Hosoyamada M. Macrophage migration inhibitory factor is a possible candidate for the induction of microalbuminuria in diabetic db/db mice. Biological and Pharmaceutical Bulletin. 2013;36(5):741-7. 10.1248/bpb.b12-00741 23649333

[28] Rasanayagam L.J., Lim K.L., Beng C.G., K.S. L. Measurement of urine albumin using bromocresol green. Clin Chim Acta. 1973;44:53-7. 10.1016/0009-8981(73)90159-9 4707638

[29] De Jong J, Wevers R, Laarakkers C, Poorthuis B. Dimethylmethylene blue-based spectrophotometry of glycosaminoglycans in untreated urine: a rapid screening procedure for mucopolysaccharidoses. Clinical chemistry. 1989;35(7):1472-7. 2503262

[30] Janssen U, Riley SG, Vassiliadou A, Floege Jr, Phillips AO. Hypertension superimposed on type II diabetes in Goto Kakizaki rats induces progressive nephropathy. Kidney international. 2003;63(6):2162-70. 10.1046/j.1523-1755.2003.00007.x 12753303

[31] Yung S, Chau MKM, Zhang Q ZC, Chan TM. Sulodexide Decreases Albuminuria and Regulates Matrix Protein Accumulation in C57BL/6 Mice with Streptozotocin-Induced Type I Diabetic Nephropathy. PLoS ONE. 2013;8(1): e54501. 10.1371/journal.pone.0054501 PMC3551764 23349910

[32] Vanden Born J, van Kraats AA, Hill S, Bakker MA, Berden JH. Vessel wall heparan sulfate and transcapillary passage of albumin in experimental diabetes in the rat. Nephrol Dial Transplant. 1997;12(2):27-31. 9269696

[33] Reddi AS, Ramamurti R, Miller M, Dhuper S, Lesker N. Enalapril improves albuminuria by preventing glomerular loss of heparan sulfate in diabetic rats. Biochem Med Metab Biol. 1991;45(119-131). 10.1016/0885-4505(91)90014-c 2015105

[34] Dilek Gogas Yavuz, Halil Onder Ersoz, Morvet Tuncel, Mustafa F Sargon, Belgin Kookkaya, Ahiskali R, et al. Effects of Aminoguanidine on Glomerular Basement Membrane Thickness and Anionic Charge in a Diabetic Rat Model. Int Jnl Experimental Diab Res. 2001;2:225-32. 10.1155/EDR.2001.225 PMC2478545 12369711

[35] Zhigang Wang, Meng Wei, Meng Wang, Lei Chen, Hua Liu, Yi Ren, et al. Inhibition of Macrophage Migration Inhibitory Factor Reduces Diabetic Nephropathy in Type II Diabetes Mice. Inflammation,. 2014;37(6). 10.1007/s10753-014-9934-x 24958012

[36] Al-Abed Y, S. VanPatten. MIF as a disease target: ISO-1 as a proof-of-concept therapeutic. Future Medicinal Chemistry. 2011;3(1):45-63. 10.4155/fmc.10.281 21428825

[37] You H, T. Gao, T.K. Cooper, W. Brian Reeves, A.S.Awad. Macrophages directly mediate diabetic renal injury. Renal Physiology. 2013;305(12):1719-27. 10.1152/ajprenal.00141.2013 PMC3882451 24173355

